# Multicore-shell nanofiber architecture of polyimide/polyvinylidene fluoride blend for thermal and long-term stability of lithium ion battery separator

**DOI:** 10.1038/srep36977

**Published:** 2016-11-11

**Authors:** Sejoon Park, Chung Woo Son, Sungho Lee, Dong Young Kim, Cheolmin Park, Kwang Sup Eom, Thomas F. Fuller, Han-Ik Joh, Seong Mu Jo

**Affiliations:** 1 Carbon Composite Materials Research Center, Institute of Advanced Composite Materials, Korea Institute of Science and Technology, Jeollabuk-do, 55324, Korea; 2 Center for Materials Architecting, Korea Institute of Science and Technology, Hwarang-Ro 14gil-5, Seongbuk-Gu, Seoul 136-791, Republic of Korea; 3 Department of Materials Science and Engineering, Yonsei University 50 Yonsei-Ro, Seodaemun-Gu, Seoul 120-749, Republic of Korea; 4 School of Materials Science & Engineering (SMSE), Gwangju Institute of Science & Technology (GIST), Cheomdangwagiro 123 Gwangju, 61005, South Korea; 5 School of Chemical & Bimolecular Engineering, Georgia Institute of Technology, Atlanta, Georgia 30332, USA

## Abstract

Li-ion battery, separator, multicoreshell structure, thermal stability, long-term stability. A nanofibrous membrane with multiple cores of polyimide (PI) in the shell of polyvinylidene fluoride (PVdF) was prepared using a facile one-pot electrospinning technique with a single nozzle. Unique multicore-shell (MCS) structure of the electrospun composite fibers was obtained, which resulted from electrospinning a phase-separated polymer composite solution. Multiple PI core fibrils with high molecular orientation were well-embedded across the cross-section and contributed remarkable thermal stabilities to the MCS membrane. Thus, no outbreaks were found in its dimension and ionic resistance up to 200 and 250 °C, respectively. Moreover, the MCS membrane (at ~200 °C), as a lithium ion battery (LIB) separator, showed superior thermal and electrochemical stabilities compared with a widely used commercial separator (~120 °C). The average capacity decay rate of LIB for 500 cycles was calculated to be approximately 0.030 mAh/g/cycle. This value demonstrated exceptional long-term stability compared with commercial LIBs and with two other types (single core-shell and co-electrospun separators incorporating with functionalized TiO_2_) of PI/PVdF composite separators. The proper architecture and synergy effects of multiple PI nanofibrils as a thermally stable polymer in the PVdF shell as electrolyte compatible polymers are responsible for the superior thermal performance and long-term stability of the LIB.

The lithium ion battery (LIB) is the most popular energy storage device. Currently, LIBs have been attracting significant attention compared with any time in the past due to the electrification of transportation^1^. Therefore, the development of three LIB elements of LIB (i.e., cathode, anode and separator) is addressed to develop LIBs with a high capacity, a high power density, a high safety level and a long-term cycle life. As far as the safety and long-term cycle life are concerned, the separator, a physical barrier between the two electrodes, has been studied the most among the three elements. The thermal stability of the separator in high power battery applications is critical for preventing the initiation of thermal runaway and battery combustion via thermal melting/deformation^2^,^3^,^4^. Polyolefin separators, such as polyethylene (PE) and polypropylene (PP), begin to lose their dimensional sustainability and various functions by shut-down functionality at temperatures above 120 °C^5^,^6^,^7^. However, this safety level is not effective for high power batteries when multiple cells are connected in series^8^. The non-shutdown separator with high temperature durability is more appropriate for the safety of high power batteries. Previous work has explored new approaches by introducing new polymer materials, an electrode/separator assembly^9^, polymer structure modifications^10^,^11^, composite membranes with organic or inorganic materials^12^,^13^,^14^,^15^, and surface coatings^16^,^17^ on the separators. However, these suggestions had critical disadvantages such as complicated preparation processes, impurities observed during cross-linking or grafting, and low processability during the cell preparation.

In previous studies, an electrospun polyvinylidene fluoride (PVdF)- or polyacrylonitrile (PAN)-based fibrous membrane was used for the LIB separator because of its high porosity, high ionic conductivity, and wide electrochemical stability window^18^,^19^,^20^,^21^,^22^,^23^. Thus, while good performance of their prototype cells was found in terms of discharge capacity and cycle performance at temperatures as low as 60 °C, the dimensional stability of the separator was not maintained due to swelling from electrolyte solution, which corresponded to low mechanical properties (<8 MPa strength, <30 MPa modulus, PVdF, Kynar 761)^18^ and fading of capacity after several hundred cycles. Additionally, PVdF and P(VdF-hexafluoropropylene) have inappropriately low thermal stabilities at high temperatures to be used in high power LIBs in electrical vehicles. To compensate for the drawbacks due to one type of component, bi-component nanofibers with a core-shell structure that are prepared using electrospinning technique have been widely studied because of their application in mechanically reinforced scaffolds, mesoporous separating membranes, drug delivery systems, and highly sensitive sensors^24^,^25^. A conventional preparation method (i.e., a coannular nozzle where two types of feeds from multiple feedlines are simultaneously spun) was used to prepare these nanofibers with a core-shell structure.

In this study, to improve the thermal and long-term stability of the LIB separator, a PI-reinforced PVdF-based separator was prepared using simple electrospinning of one type of solution with two immiscible polymers through a single nozzle. The PI component was composited in a unique form of multiple core strings in a PVdF fiber, which resulted in a nano multicore-shell (MCS) architecture. The multiple PI core strings embedded along the PVdF fiber axis were derived from multiple PI droplets dispersed in dope during the spinning process. The method is simple and effective for distributing PI reinforcements across the fiber cross section because it does not require a special nozzle feed from two different types of dope for constructing multiple PI core strings. It requires only one single nozzle feed from a single type of dope. Compared with the other two electrospun PI/PVdF composite separators with different architectures (a single core-shell and co-electrospun separator incorporating functionalized TiO_2_) that were derived from multiple types of feedlines^13^,^26^, the MCS PI/PVdF separator prepared using a single nozzle had the best long-term stability in terms of the average capacity decay rate. The feasibility of PI droplet formation and deformation into core fibrils during the process were studied using a phase field theory. The surface morphology and internal microstructure of the electrospun PI/PVdF MCS composite membrane were investigated. Additionally, the thermal stability of the PI/PVdF MCS membrane and its conductivity cell were evaluated in the temperature region of 40–250 °C. The ionic resistivity (below ~5 ohm/cm up to 250 °C), ionic conductivity (1.3 × 10^−3^ Scm^−1^), interfacial resistance, and electrochemical impedance were measured as functions of temperature for both the conductivity and prototype cells to investigate the thermal properties of the MCS separator for high temperature applications. The battery performance of the prototype cell, including the long-term cycle performance and temperature dependence of discharge capacity, was evaluated.

## Experimental procedures

### Preparation of a PI/PVdF mixed solution

The polymer solution was prepared from 6.5 wt.% poly(vinylidene fluoride) (PVdF, Atofina, Kynar 761) and 6.5 wt.% polyimide (PI, Ciba Specialty, Matrimid 5218) in a mixed solvent system containing dimethylacetamide (DMAc)/tetrahydrofuran (THF) (6/4, wt. ratio). The polymer materials were dried under vacuum at 80 °C to eliminate any moisture and volatile impurities before the polymer solution was prepared. HPLC-grade solvents (Merck) were used in the electrospinning experiments without any further purification. The polymer materials were added to the mixed solvents, and then, the mixture was stirred at 80 °C to prepare the homogeneously dispersed polymer solution. The PE separator (SKC provided, South Korea) was used as the reference material.

### Preparation and post-treatments of the PI/PVdF MCS composite membranes

The nanofibrous PI/PVdF MCS composite membrane was prepared using the single nozzle electrospinning method that was described in detail in earlier studies^21^,^22^. An applied voltage of 10.5 kV, a tip-grounded collector distance of 14 cm, a needle size of 30 G, and a solution feeding rate of 30 μl/min were the parameters used. Post-processing (including hot pressing at 150 °C, heat stretching at 150 °C and then post-heat treatment at 170 °C) was on the electrospun membrane to control its thickness and porosity. The membrane with a thickness of approximately 20 μm was obtained after post-processing. This post-processed membrane was dried in a vacuum oven at 80 °C to remove any remaining solvents and was then stored in a glove box filled with argon gas (H_2_O < 1 ppm) prior to use.

### Characterization of the physical properties of the PI/PVdF MCS composite separator

The surface morphology of the PI/PVdF MCS separator was measured using a field-enhanced scanning electron microscope (FE-SEM, Hitachi, S-4200) under vacuum. All samples were gold-coated prior to SEM measurements. The porosity and electrolyte uptake measurements of the MCS1 separator and of the PE separator were measured by soaking the separators in an electrolyte solution of 1 M LiPF_6_-EC/PC/DEC/VC (35.4/17.2/45.1/2.3, wt.%). The mechanical properties of both separators were measured using a universal testing instrument (Instron, Model 4464) with a cross-head speed of 10 mm/min at room temperature. Each sample was cut into 6.0 cm × 0.5 cm strips, and their thicknesses were measured using a dial gauge (Mitsutoyo, BWB836) prior to the measurements. This test was performed according to the ASTM standard D882-95a. Additionally, the thermal shrinkage tests of the MCS1 and PE separators (3 cm × 3 cm) were conducted by storing them in an oven at 200 °C for 1 hr. The dimensional changes were evaluated by measuring the dimensions of the resulting samples.

To investigate the high temperature melt integrity (HTMI) of MCS1 and PE separators, thermo-mechanical analysis (TMA, Perkin-Elmer, TMA-7) measurements were conducted. The length change as a function of temperature was monitored in the temperature range of 50–400 °C. The samples with dimensions of 1 cm × 0.5 cm were vacuum-dried prior to use. The heating rate was 5 °C/min, and the weight of load was 200 g. The static force was 50 mN.

### Cell preparation

Two types of cells (i.e., conductivity and prototype cells) for both MCS1 and PE separators were prepared. The conductivity cells were assembled by sandwiching the separators between the two blocking stainless steel electrodes. The polymer electrolyte was prepared by soaking the separator in an electrolyte solution of 1 M LiPF_6_-EC/PC/DEC/VC (35.4/17.2/45.1/2.3 wt.%) at room temperature in a glove box filled with argon gas. The prototype cell was prepared by assembling three components, i.e., the polyelectrolyte, a mesocarbon microbead (MCMB) anode (SKC supplied, Korea) and a LiCoO_2_ cathode (SKC supplied, Korea) in a vacuum-sealed aluminum-plastic pouch. The cell size was 3 cm × 4 cm, and its theoretical capacity was 141 mAh/g. The entire assembling process was performed in a glove box filled with argon gas (H_2_O < 1 ppm).

### Electrochemical Characterization

A multichannel potentiostat equipped with impedance modules (BioLogic) was used to measure electrochemical impedance at 1 kHz in the 100 mHz–1 MHz frequency range with an AC voltage of 10 mV. The conductivity cells were heated at a rate of 5 °C/min from 40 to 250 °C. After reaching the desired temperature, a 5 min rest period was applied before the impedance measurements were recorded every 10 °C.

The prototype cells were charged to 50% SOC and then were heated at a rate of 5 °C/min from 40 to 250 °C. The rest time was 5 min for every 10 °C, and the impedance was measured from 100 mHz to 1 MHz for 150 seconds, along with the OCV for 10 seconds and the impedance at 1 kHz for 10 seconds.

The temperature dependence of the discharge capacity of the prototype cells was investigated, as follows. The cells were charged at C/10 and then were discharged at 1C with cut-off voltages of 2.75 and 4.2 V at intervals of 20 °C in the temperature range of 0–80 °C. Additionally, the cycle performance of prototype cells was examined using a battery cycler (WBCS3000, WonATech Co.) at room temperature. The cell performance was galvanostatically evaluated with cut-off voltages of 2.75 and 4.2 V at a 1C/1C rate.

## Results and Discussion

**Figure 1:**
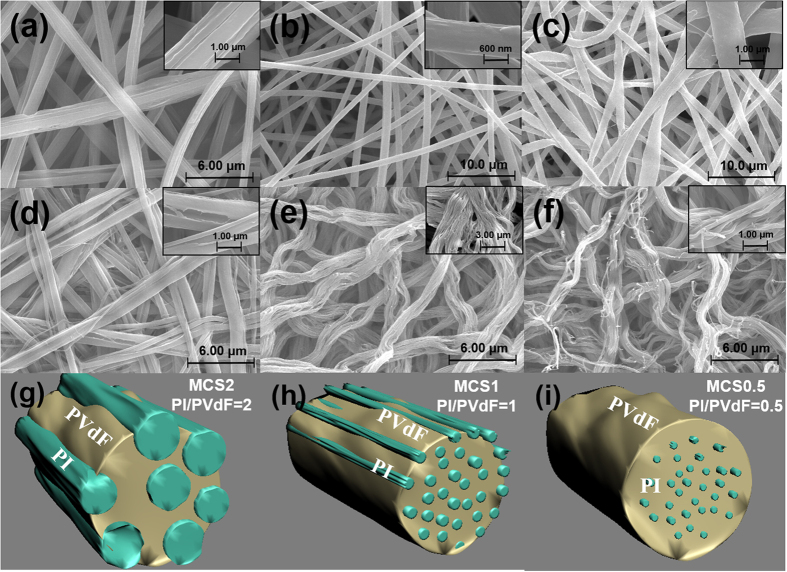
SEM images of the electrospun PI/PVdF MCS composite nanofibrous membrane, residual PI core fibrils after solvent extraction, and the schematic of the multicore-shell structure (**a**,**d**,**g**) MCS2, PI:PVdF = 2:1 wt. ratio, (**b**,**e**,**h**) MCS1, PI:PVdF = 1:1 wt. ratio, and (**c**,**f**,**i**) MCS0.5, PI:PVdF = 1:2 wt. ratio. The winkles along the electrospun fiber become severe, and the diameter of PI core fibrils tends to increase as the PI content is increased.

Figure 1 shows schematic and SEM images of single fiber morphologies in electrospun PI/PVdF MCS membranes as functions of mixing weight ratio of polymers. The surface morphology was smoother with an increase in the PVdF content in the PI/PVdF composite solution. The morphology of the membrane with a ratio of 2 (MCS2, PI/PVdF = 2), which was prepared using a PI-rich composite solution, was an uneven surface with wrinkles along the fiber axis, as shown in Fig. 1a. Slight wrinkles on a fiber surface were found in MCS1 (PI/PVdF = 1), as shown in Fig. 1b. However, in the case of the MCS0.5 (PI/PVdF = 1/2), a smooth fiber morphology was obtained, which was similar to the pure PVdF membrane case reported in our previous studies^22^,^23^ (Fig. 1c).

To observe the internal microstructure of MCS membranes, the PVdF component was selectively removed using solvent extraction with acetone. The PVdF part within the MCS membranes was dissolved in acetone during the extraction, and the PI part remained without any loss due to its insolubility in acetone. Interestingly, the internal microstructure as a function of the polymer ratio was different from the apparent surface morphologies. Figure 1d shows the MCS2 membrane after the PVdF component was removed. The morphology of the remaining PI fibers was similar to the wrinkles that formed along the fiber axis in the as-electrospun MCS2 fibers in Fig. 1(a). Therefore, it is believed that the as-electrospun MCS2 membrane was composed of ultrafine PI fiber bundles with diameters of 300–500 nm and the PVdF component acted as a binder for this bundle. In the case of the MCS1 and MCS0.5 separators, the residual PI fibers were observed as ultrafine fibrils with diameters of 100–200 nm, as shown in Fig. 1(e,f). The PI fibers in MCS1 and MCS0.5 were much fully embedded within PVdF, and the morphology of MCS fibers was smooth unlike that of MCS2. Therefore, the morphology and composition of MCS fibers were easily controlled as a function of mixing weight ratio, as illustrated in Fig. 1(g–i).

To understand the MCS structure formation mechanism, a theoretical analysis of the phase separation process in the dope and shear-induced deformation was conducted. The bulk phase separation of the mixed polymer solution occurred resulted in both circular droplets and the continuous matrix form. This result occurred because the PI- and PVdF-rich phases were completely isolated under our experimental conditions. The two phases were mixed relatively homogenously during the initial mixing step. As time progressed, multiple small PI droplets were formed and coalesced into large droplets (Figure S1). The coalescence between droplets led to an increase in size and simultaneous decrease in the number of droplets. The droplets started to transform into core strings when the droplets were subjected to flow through the syringe capillary during electrospinning (Figure S2). Considering the molecular weight of the polymers, Flory-Huggins interaction parameters (χ), component ratios, and calculated viscosities of the droplets and other parts, the PI droplets were highly stretched compared with the PVdF parts within the mixture. This led to the formation of multiple ultrafine strings and outer shells with PI and PVdF polymers, respectively, as described in the Supporting Information. Therefore, it is believed that the intrinsic immiscibility (χ_PI-PVdF_ = 0.37) of the polymers can form a novel MCS membrane using single-nozzle electrospinning.

**Figure 2:**
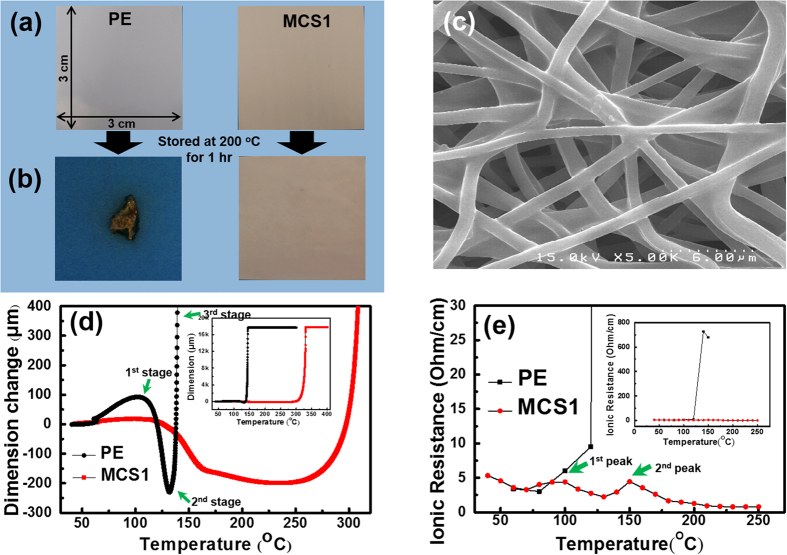
Photos of the MCS1 and the PE separator (**a**) before and (**b**) after the thermal stability oven test. The original dimension of each membrane before the test was 3 cm × 3 cm. (**c**) SEM image of the MCS1 after the thermal stability oven test. The network and pore structures were maintained. (**d**) The differential HTMI of the MCS1 separator and the PE separator. The inset is the original HTMI. Note the following three stages of dimensional change: i) short elongation, ii) slight shrinkage and ii) complete elongation. (**e**) Temperature dependence of ionic resistivity in the SUS/membrane/SUS conductivity cells with the PE and the MCS1 separator in 1 M LiPF_6_-EC/PC/DEC/VC. The inset is a large-scale plot. Notice the two peaks of the MCS separator, which correspond to the melting points of the swollen polymers on the surface of the fiber (90 °C) and the partially swollen PVdF in the core region of the fibers (150 °C).

The thermal shrinkage test was conducted through storage at 200 °C for 1 hr to verify the effect of the MCS structure on thermal stability. Figure 2a,b shows photos of the PE separator and of the MCS1 before and after the thermal shrinkage test. The commercial PE separator significantly shrank because the melting temperature (T_m_) of the PE is 126.4 °C. Interestingly, no noticeable dimensional changes in MCS1 was found at the temperature of 200 °C even though the partially melted surface of the MCS1 separator after the test was observed due to PVdF (T_m_ = 165 °C), as shown in Fig. 2c. Thus, the thermally stable PI (T_m_ = 388 °C) multicore fibrils that aligned along the PVdF shell could provide the dimensional and porous maintenance of the MCS separator at 200 °C.

The thermal stability, including thermally induced shrinkage or elongation of the membrane, was also investigated by measuring the high temperature melt integrity (HTMI) using thermo-mechanical analysis (TMA). In general, there are three stages of dimensional changes: a short elongation during the initial stage, a slight shrinkage during the second stage, and then, another long elongation along the loading axis (third stage). The PE separator showed a greater elongation than the MCS1 separator during the initial stage, a steep shrinkage during the second stage, and then, a drastic elongation at approximately 140 °C when the PE melted. However, a high dimensional stability (~300 °C) was obtained except for an insignificant elongation during the initial stage and a mild shrinkage during the second stage.

The conductivity cells with either MCS1 or PE separators were assembled for an ionic resistance test by sandwiching the separator between the two blocking stainless steel electrodes (SUS/separator/SUS) as a function of temperature. For the PE separator, the ionic resistivity drastically increased at approximately 120 °C because the PE separator melted. This type of behavior is the thermal shutdown behavior of the PE separator, which is similar to its melting properties. However, in the case of the MCS separator, no significant increase in the ionic resistance was observed up to 250 °C, with the exception of two small peaks were observed at approximately 90 and 150 °C. In our previous study, the differential scanning calorimetry curve of a separator with only PVdF contained two major peaks, which corresponded to the melting points of swollen polymers on the surface of the fiber (90 °C) and the partially swollen PVdF in the core region of the fiber (135 °C)^22^. In contrast to a previous study, there were no significant changes in the resistance and in the observation of a second peak at a higher temperature. Therefore, it is believed that the existence of multiple PI core fibrils along the fiber positively shifted the melting temperature of the inner region of PVdF, which regulated swelling and the thermal behavior of the separator and led to the thermal stability with respect to the maintainability of the dimension and resistance at high temperatures or during severe operating conditions.

**Figure 3:**
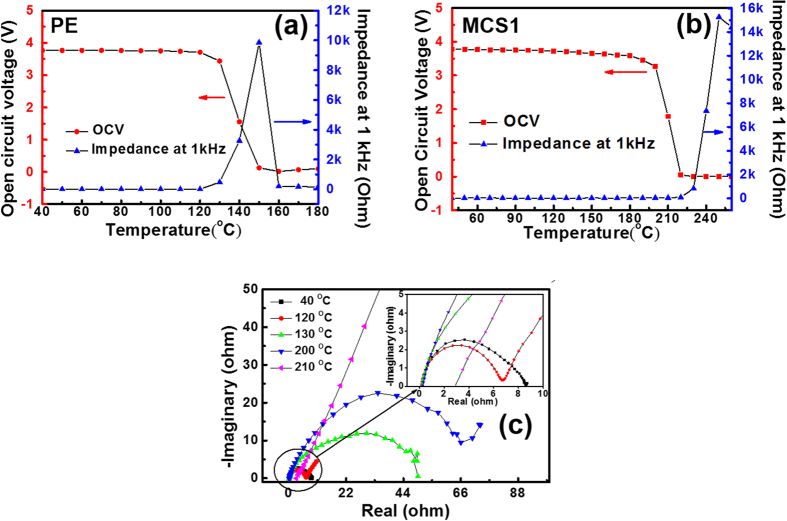
Impedance at 1 kHz and open circuit voltage (OCV) of prototype cells with (**a**) MCS1 and with (**b**) the PE separator in 1 M LiPF_6_-EC/PC/DEC/VC. (**c**) Impedance spectra as a function of temperature of the prototype cells with the MCS1. The sharp increase in the interfacial resistance (R_int_) is observed at temperature between 200 and 210 °C for electrode decomposition.

The thermal behavior of open circuit voltage (OCV) and the impedance at 1 kHz were measured in the temperature range of 40–250 °C to investigate the thermal stability of the MCS1 and PE separator in a LIB cell with LiCoO_2_ as the cathode and mesocarbon microbeads as the anode. Both cells were charged to the 50% state of charge for each measurement. The LIB cell with the PE separator showed lower thermal stability than the cell with the MCS1 separator. The OCV of the LIB cell with the PE separator gradually decreased at 120 °C and was approximately zero at 140 °C, as shown in Fig. 3a. Additionally, the AC impedance at 1 kHz steeply increased at 130 °C. As expected from the ionic resistance test in a conductivity cell, the melting of the PE separator was responsible for the two outbreaks, which corresponded to the sharp increase in ionic resistance at approximately 120 °C (Fig. 2e). In the cell with the MCS1 separator, the AC impedance sharply increased at 220 °C and OCV steeply decreased at 200 °C and reached a value of approximately 0 V at ~220 °C (Fig. 3b). The outbreak temperatures measured in the cell were lower than those in the ionic resistance cell due to the lower thermal stability of the LiCoO_2_ cathode. It is well known that LiCoO_2_ in LIBs thermally decomposed through O_2_ release at approximately 200 °C, and the electrolyte solution degraded the electrode surface^27^,^28^,^29^. Thus, this side reaction could have caused the sharp increase in the AC impedance of the cell with the MCS1 separator.

Figure 3c shows the impedance spectra of LIB cells with the separators as a function of temperature. The bulk resistance (R_b_) of the MCS1 separator with the electrolyte solution was approximately constant up to 200 °C but slightly increased at 210 °C. However, the interfacial resistance (R_int_) between the MCS1 separator and the electrode was only a few tens of ohms until 200 °C, and then, sharply increased at 210 °C. Based on the fact that there was no significant increase in ionic resistance of the conductivity cell up to 250 °C (Fig. 2e), an unstable interface was formed via electrode decomposition, which contributed to the sharp increase in the AC impedance at 1 kHz and to the steep drop in the OCV (Fig. 3b). These results confirmed that the MCS1 separator had a high thermal stability in the LIB at temperatures greater than 200 °C. Additionally, the thermal stability in the LIB cell was lower than in the conductivity cell. This was attributed to electrode decomposition and/or electrolyte solution degradation at the interface between the electrode and polymer electrolytes. The thermal failure of the MCS1 separator was not a factor.

**Figure 4:**
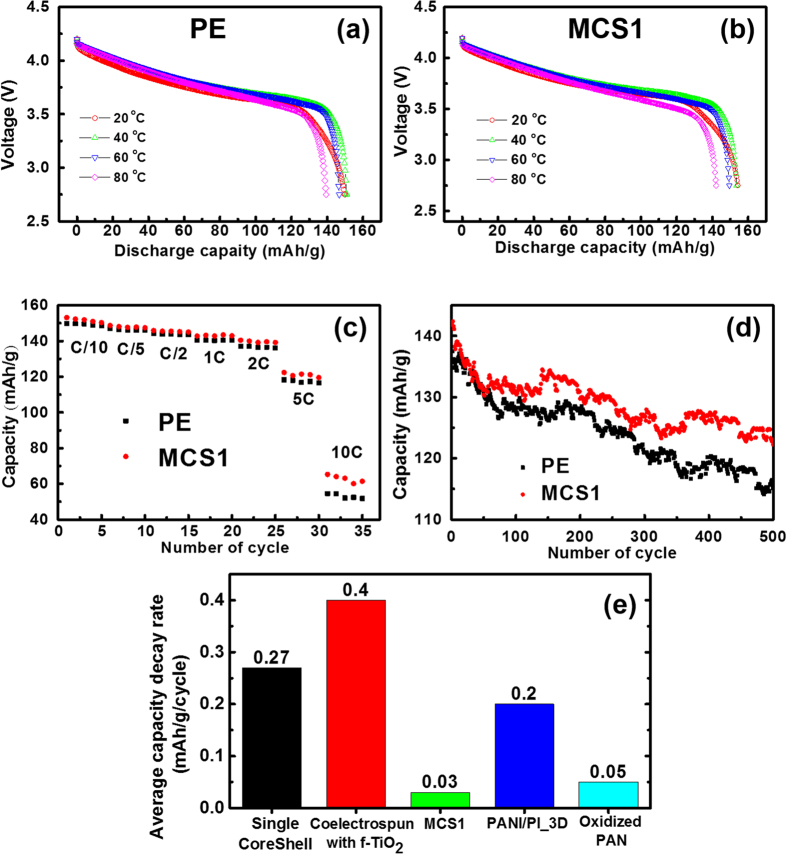
Temperature dependence of the discharge capacity in the prototype cells with (**a**) the PE separator and with (**b**) the MCS1 separator in 1 M LiPF_6_-EC/PC/DEC/VC. (**c**) C-rate performance of prototype cells with the MCS1 composite nanofibrous membrane. (**d**) Long-term cycle performance of the prototype cells with the PE separator and MCS1 in 1 M LiPF_6_-EC/PC/DEC/VC at a charge/discharge rate of 1.0C/1.0C. (**e**) The average capacity decay rate comparison of other PI/PVdF composite Li-ion battery separators (single core-shell, co-electrospun) and two other separators of different materials (polyaniline/polyimide composite with hierarchical 3D micro/nano-architecture, PANI/PI_3D, and partially oxidized polyacrylonitrile, Oxidized PAN). The long-term cycle performance for both single core-shell and co-electrospun was conducted at a charge/discharge rate of 0.5C/0.5C. The average capacity decay was calculated by dividing the difference of start and end capacities by the total cycle number.

The discharge capacities of LIB cells with both the MCS1 separator and the PE separator in 1 M LiPF_6_-EC/PC/DEC/VC (35.4/17.2/45.1/2.3, wt.%) were measured in the 20–80 °C temperature region. In Fig. 4a,b, higher discharge capacities were found in the cell with MCS1 separator than the cell with the PE separator in the operating temperature range. A higher electrolyte uptake of 427% was found for the MCS1 separator compared with the value of 203% for the PE separator. This difference was due to the interaction between the PVdF component on the surface and the solvent molecules of the electrolyte solution^18^, as summarized in Table S1. Considering the ionic resistance and electrolyte uptake, the control of the structure and morphology of the MCS1 separator affects the LIB performance. However, the difference in the discharge capacities becomes indefinite at the high temperature region because the cell temperature increase is due to the external heat source. The c-rate performance test on the two cells for 5 cycles at seven different c-rates was performed (Fig. 4c). The prototype cell with the MCS1 separator always had a higher capacity for all c-rates compared with the PE separator cell. Additionally, the capacity difference became more significant at high charge rates. The capacity difference between the cells gradually began to increase at 1C and was clearly observed at the 10C charge/discharge rate. The enormous differences were responsible for the internal heat that was generated within the cell. The internal heat generation became significant at high discharge rates due to the ohmic drop and polarization effects^29^,^30^,^31^. Therefore, the MCS1 separator possessed superior performance compared with the PE separator in terms of the internal and external thermal management.

The long-term cycle performance (500 cycles) was examined at a charge/discharge rate of 1.0C/1.0C, as shown in Fig. 4d. The cycle performances during 500 cycles were stable for both the prototype cells with the MCS1 and the PE separator. However, the cell with the MCS separator performed better cycle stability than the PE separator cell. In the case of the cell with the MCS separator, an initial discharge capacity of 141 mAh/g was measured, and 89.3% of the initial discharge capacity was maintained after 500 cycles. However, in the case of the PE separator cell, a discharge capacity of 137.5 mAh/g during the first cycle was found, and 83.6% of the initial discharge capacity after 500 cycles was retained. The average capacity decay rate of the two electrospun PI/PVdF composite separators with two different architectures (single core-shell and co-electrospun incorporating functionalized TiO_2_)^13^,^26^ and other two thermally stable separators of different materials (polyaniline/polyimide composite with hierarchical 3D micro/nano-architecture, PANI/PI_3D, and partially oxidized polyacrylonitrile, Oxidized PAN)^32^,^33^ are plotted with the average capacity decay rate of the MCS1 separator in Fig. 4e to compare the long-term cycle performance. The MCS1 separator was marked as the lowest average decay rate of 0.030 mAh/g/cycle among the three types of PI/PVdF composite separators and showed superior long-term stability for appropriate architecture. Also, compared with other separators of different materials, the MCS separator showed notable long-term stability. It was concluded that the MCS separator was more beneficial in suppressing the growth of dendrites and in maintaining its uniform permeability and thickness^5^.

## Conclusions

The porous PI/PVdF composite separator, which was composed of nanofibers with a multicore-shell (MCS) structure, was prepared using a facile electrospinning technique with a single nozzle. The intrinsic immiscibility of polymers allowed for the formation of a novel MCS membrane despite single-nozzle electrospinning. The unique MCS separator showed better thermal stability, mechanical properties, and long-term battery performance than a commercial PE separator due to its well-distributed thermally stable PI nanofibrils in a PVdF shell and external PVdF and electrolyte interactions. Therefore, the MCS separator prepared using single-nozzle electrospinning is a promising material to be used as a thermally stable and active separator for Li-based batteries.

## Additional Information

**How to cite this article**: Park, S. *et al*. Multicore-shell nanofiber architecture of polyimide/polyvinylidene fluoride blend for thermal and long-term stability of lithium ion battery separator. *Sci. Rep.* **6**, 36977; doi: 10.1038/srep36977 (2016).

**Publisher’s note:** Springer Nature remains neutral with regard to jurisdictional claims in published maps and institutional affiliations.

## Supplementary Material

Supplementary Information
