# Supplementary material for: Multicore-shell nanofiber architecture of polyimide/polyvinylidene fluoride blend for thermal and long-term stability of lithium ion battery separator
Source: Sci Rep. 2016 Nov 11;6:36977. doi: 10.1038/srep36977 (PMC5105080; doi:10.1038/srep36977)
Supplement: Supplementary Information [file srep36977-s1.pdf]

Multicore-shell nanofiber architecture of polyimide/polyvinylidene fluoride blend for thermal and long-term stability of lithium ion battery separator

Sejoon Park,[§,▲] *Chung Woo Son,*[§,▲] *Sungho Lee,*[§] *Dong Young Kim,*[‡] *Cheolmin Park,*[†] *Kwang Sup Eom,*[∥] *Thomas F. Fuller,*[∥] *Han-Ik Joh,*[§,*] *Seong Mu Jo*[§,*]

[§]Carbon Composite Materials Research Center, Institute of Advanced Composite Materials, Korea Institute of Science and Technology, Jeollabuk-do, 55324, Korea

[‡]Center for Materials Architecting, Korea Institute of Science and Technology Hwarang-Ro 14gil-5, Seongbuk-Gu, Seoul 136-791, Republic of Korea

[†]Department of Materials Science and Engineering, Yonsei University 50 Yonsei-Ro, Seodaemun-Gu, Seoul 120-749, Republic of Korea

[∥]School of Chemical & Bimolecular Engineering, Georgia Institute of Technology, Atlanta, Georgia 30332, USA

ASSOCIATED CONTENT

Supporting Information.

1. Study of the formation mechanism of multicore-shell structures from PI/PVdF solution via PI droplet formation and deformation.

2. Summary of physical properties of the MCS1 separator and the reference PE separator.

3. Thermal mechanical analysis and SEM images as a function of temperature

The mechanism of MCS structure construction can be divided into two steps. The first step is the nucleation and growth of multiple PI droplets in a bulk solution. The second step is their deformation into fibrils embedded in the PVdF matrix. To understand how this MCS structure is formed, theoretical analysis on phase separation and droplet deformation in capillary flow was conducted using the following method. To simulate the phase separation dynamics, a model that depends on time and space is required. The spatial distribution of volume fraction function was employed to describe the evolution of phases.

$$\frac{\partial \phi_\alpha}{\partial t} = -\boldsymbol{\nabla} \cdot \boldsymbol{J_\alpha} \qquad (1)$$

where J_α is the flux of component α induced by diffusion, random and external fields such as a hydrodynamic flow. It is assumed that the diffusion flux of each component is proportional to the spatial gradient of the chemical potential of each component. The chemical potential of each component is derived from the partial differential of free energy, F. For the polymer mixture, the free energy is given by the Flory-Huggins-deGennes theory, as follows:

$$F = k_B T/v_0 \int dV [\sum_{\alpha=0}^{M-1} \frac{\phi_\alpha}{N_\alpha} ln\phi_\alpha + \sum_{\alpha<\beta}^{M-1} \chi_{\alpha\beta}\phi_\alpha\phi_\beta + 1/2 \sum_{\alpha<\beta}^{M-1} C_{\alpha\beta}[\nabla(\phi_\alpha - \phi_\beta)]^2] \qquad (2)$$

where $C_{\alpha\beta} = \frac{a^2}{18\phi_\alpha\phi_\beta}$, $\chi_{\alpha\beta}$ is the Flory-Huggins chi interaction parameter between α and β components, N is the segment number of component α, and v_0 is the element volume ($=a^3$). The Navier-Stokes equation with the Stokes approximation was used when the hydrodynamic effect was considered:

$$\rho\frac{\partial v}{\partial t} = \boldsymbol{\nabla}\cdot(\eta\boldsymbol{\nabla}\cdot\boldsymbol{v}) - \boldsymbol{\nabla}p \qquad (3)$$

where v is the velocity, p is the pressure, η is the viscosity, ρ is the density.

The basic equation can be summarized as follows:

$$\frac{\partial\phi_\alpha}{\partial t} = -\nabla\cdot(\boldsymbol{v}\phi_\alpha) - \nabla\cdot\boldsymbol{J}_\alpha \qquad (4)$$

$$\rho\frac{\partial v}{\partial t} = \nabla\cdot(\eta\nabla\cdot\boldsymbol{v}) - \nabla\boldsymbol{p} \qquad (5)$$

$$\nabla\cdot\boldsymbol{v} = 0 \qquad (6)$$

The equations were numerically solved using the finite difference method. To simulate the PI droplet growth, the system was discretized by a number of meshes (100-by-100). The volume fraction of each mesh was randomly initialized to describe the initial homogeneity of a well-mixed solution. For all fields, including the volume fraction, chemical potential, and diffusional flux, all boundaries were set to be at periodic conditions for the bulk description on phase separation process. To simulate the PI droplet deformation in capillary flow, the initial condition was selected as one volume fraction profile with PI droplets. Two x-axis boundaries were set to have a periodic condition to track the deformation in limited window, while the two y-axis boundaries were set to have a no-slip condition.

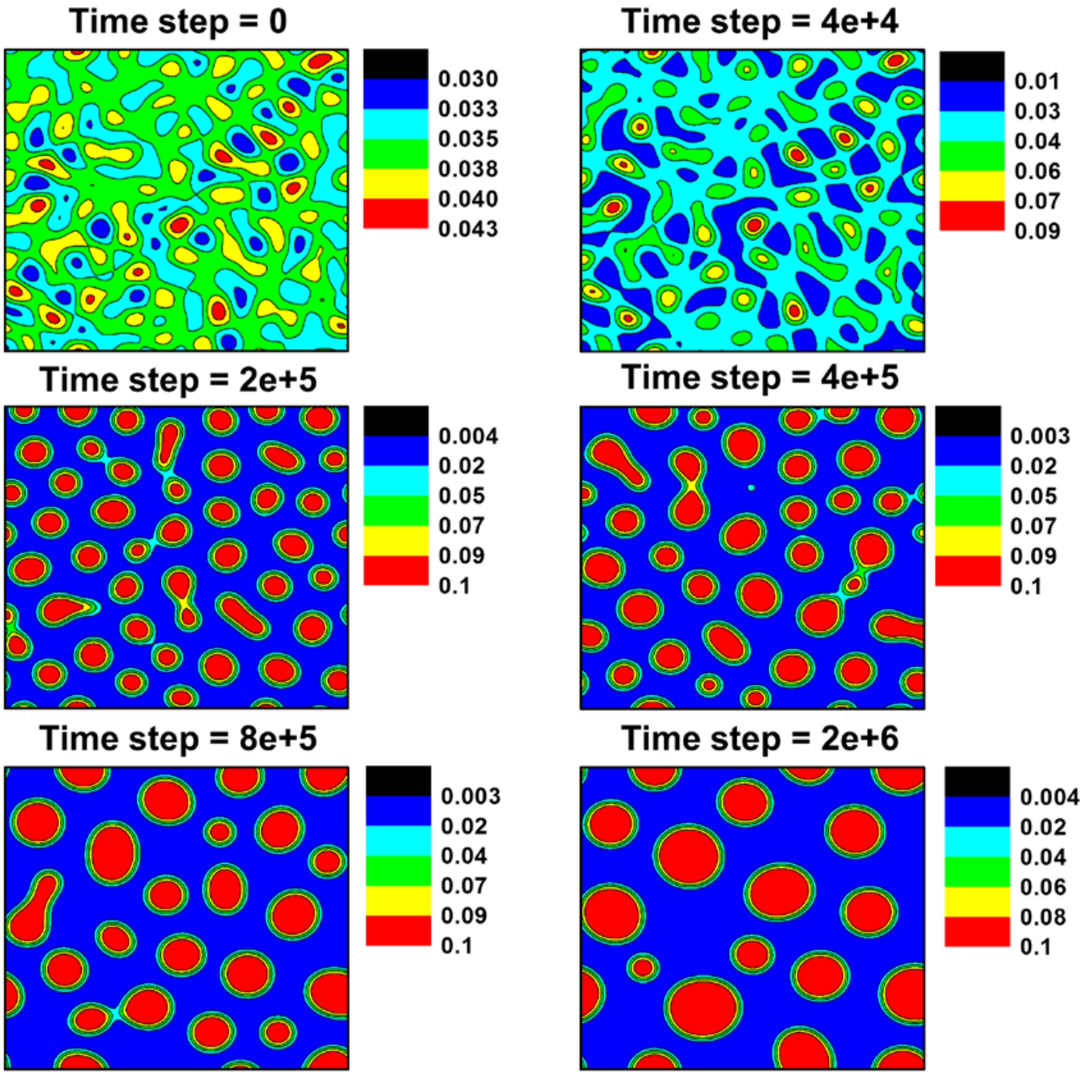

Figure S1. Simulated phase separation process of the PI-PVdF composite solution under experimental conditions. The colored bar represents the volume fraction of PI. The compete distinction between the PI-rich (Φ_{PI} = 0.11) and PI-poor (Φ_{PI} = 0.003) phases were initially formed within the time step of 120000, forming a number of PI-rich droplets dispersed in a PVdF-rich matrix. Coalescence between droplets both increases the size of droplets and decreases the number of droplets.

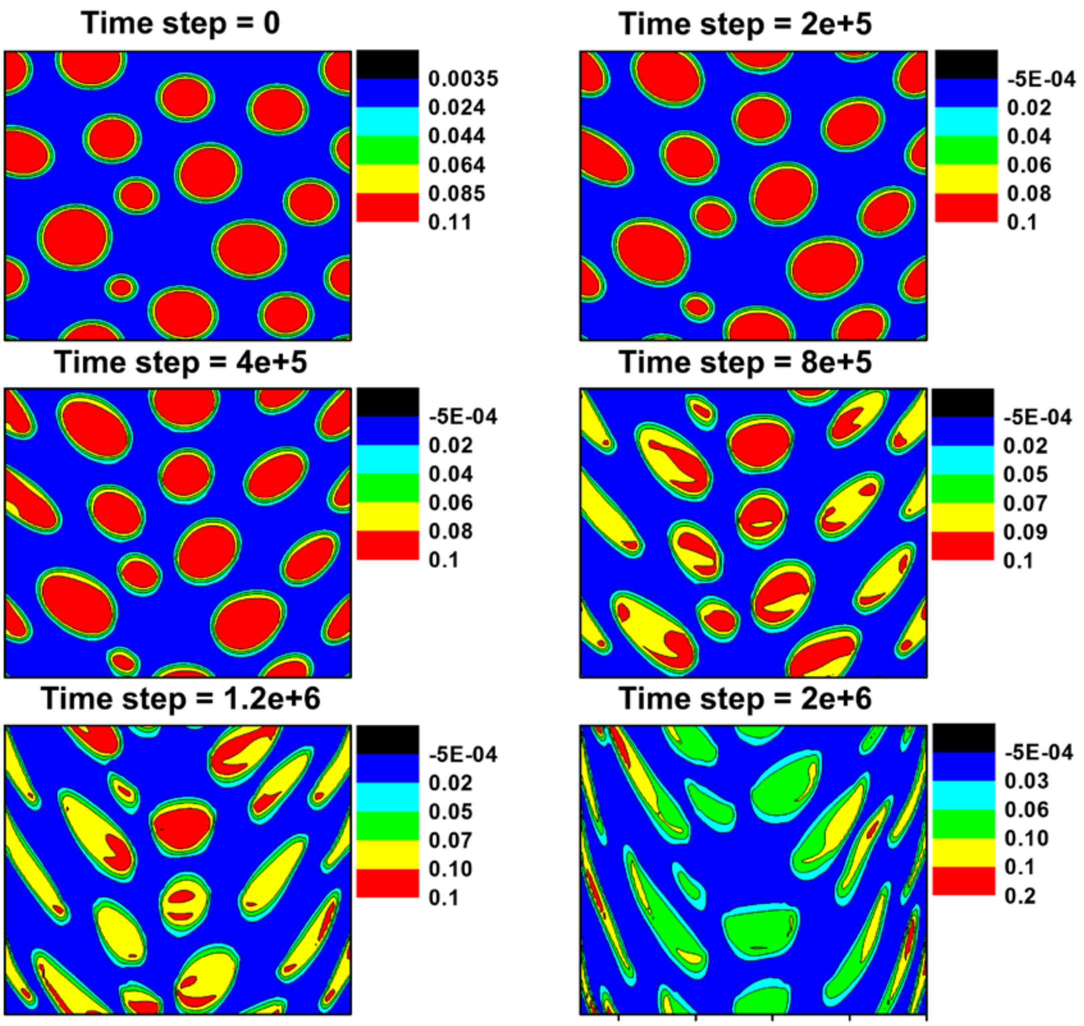

Figure S2. Simulated deformation process of PI-rich droplets under capillary flow. The colored bar represents the volume fraction of PI. The boundaries of PI droplets with the PVdF matrix are maintained in the flow. The size decrease of droplets in the vertical direction of the flow is expected to be more significant as the droplets are located near the capillary wall.

Table S1. Summary of physical properties, thermal properties, and ionic conductivities of the MCS1 and PE separators

Properties		PI/PVdF MCS1 separator	PE separator
Physical properties	Porosity by BuOH uptake, P (%)[a)]	(81) 58	60
	Electrolyte uptake, ε (%)[b)]	(657) 427	203
Mechanical properties[c)]	Max. strength (MPa)	(4.72) 24.2	83.5
	Max. strain (%)	(59.8) 10.5	112
	Modulus (MPa)	(92.4) 536	603
Thermal properties	Onset temperature (^oC)	157.3	126.4
	Crystallinity[d)] (%)	35.4	86
Ionic conductivity at 30 ^oC (S cm^{-1})		1.3×10^{-3}	0.59×10^{-3}

[a)]$P(\%) = (M_{BuOH}/\rho_{BuOH})/(M_{BuOH}/\rho_{BuOH} + M_m/\rho_p)\times100$, M_{BuOH}: mass of BuOH adsorbed, M_m: mass of the dried membrane, ρ_{BuOH}: BuOH density, ρ_p: polymer density

[b)] $\varepsilon(\%) = (M-M_0)/M_0 \times100$, M_0: mass of the membrane, M: mass of the membrane with an electrolyte solution

[c)]Values in brackets are the membrane properties prior to post-treatment

[d)] $X_c(\%)=(\Delta H_m^{sample}/\Delta H_m^*)\times100$, ΔH_m^*:104.7 J/g (PVdF), 209 J/g (PE)

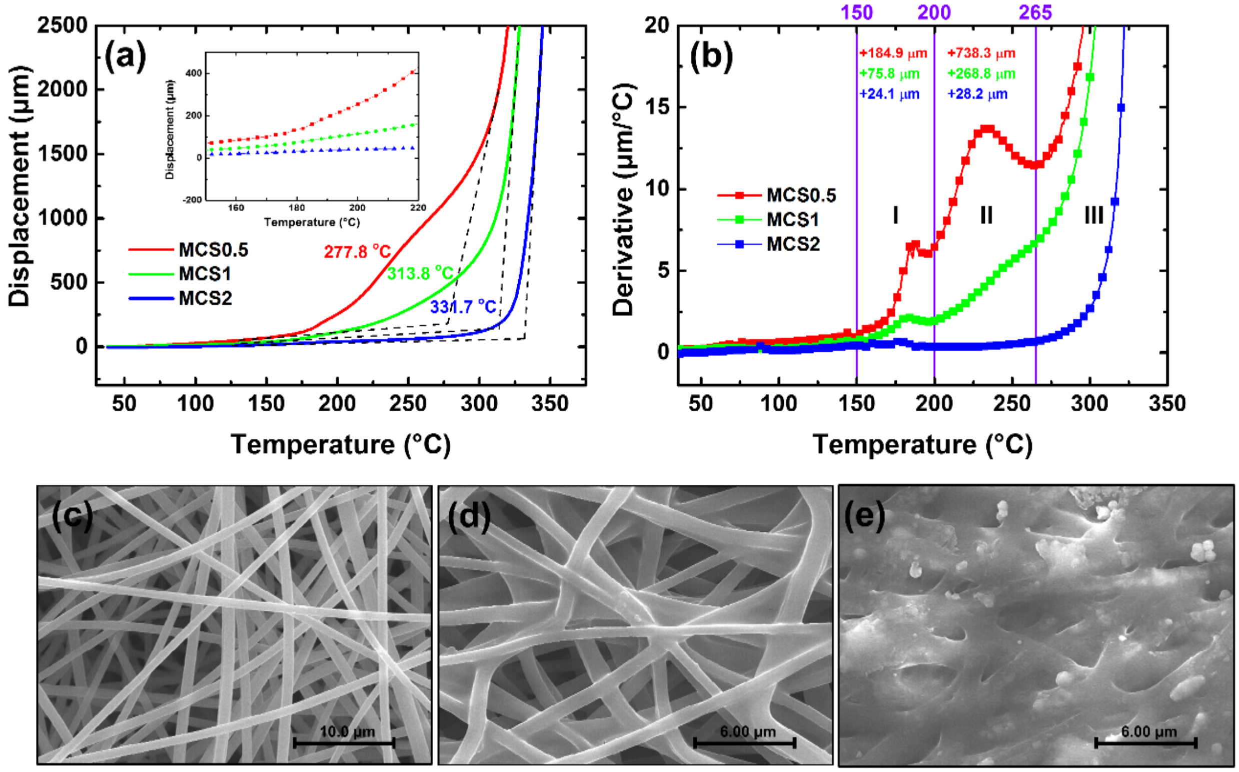

Figure S3. Structural evolution of MCS separator upon temperature ramping at 5 ^{o}C/min (35~350 ^{o}C) (a) Dimension displacement of MCS0.5, MCS1, and MCS2 under the tensile stress of 0.5 MPa; The final onset temperature of dimensional lose become lower for a separator with low PI ratio. (b) The derivative curves of dimension displacement for observing the transitions during the heating. The accumulated displacements of MCS0.5, MCS1, and MCS2 in the regions I and II are shown in corresponding colors. (c, d, e) The SEM images of MCS1 representing the visual status at each heating step.

The displacement from the original dimension of the all MCS separator was observed in the temperature range from 35 to 350 °C (Figure S3 (a) and (b)). From the derivative curves, the structural evolution can be divided into three transition regions. In region I, as the temperature reached to the melting temperature (165 °C) of PVdF, more elongation was observed for the

composite with more amount of PVdF. However, the degree of elongation was limited until 200 °C because the multicores of PI were structurally bound with shell of PVdF, leading to the improved dimensional sustainability of nanofibers. It can be easily confirmed by SEM images of the MCS separator after exposure at 200 ^{o}C (Figure S3 (d)). In region II, as the temperature become higher, the PVdF shell gradually started to melt even though the shell was reinforced by PI multicores, leading to losing their dimensional sustainability as shown in Figure S3 (e). In the final region III, PI cores were suddenly decomposed regardless of the mixing ratio and the significant elongation of the separator was observed. The final onset temperature of dimensional lose become lower for a separator with low PI ratio. The increase in PI ratio provides stronger thermal stability to MCS separator.
